# Tristetraprolin expression by keratinocytes protects against skin carcinogenesis

**DOI:** 10.1172/jci.insight.140669

**Published:** 2021-03-08

**Authors:** Assiya Assabban, Ingrid Dubois-Vedrenne, Laurye Van Maele, Rosalba Salcedo, Brittany L. Snyder, Lecong Zhou, Abdulkader Azouz, Bérengère de Toeuf, Gaëlle Lapouge, Caroline La, Maxime Melchior, Muriel Nguyen, Séverine Thomas, Si Fan Wu, Wenqian Hu, Véronique Kruys, Cédric Blanpain, Giorgio Trinchieri, Cyril Gueydan, Perry J. Blackshear, Stanislas Goriely

**Affiliations:** 1 Institute for Medical Immunology, ULB Center for Research in Immunology, and ULB Center for Cancer Research, Université Libre de Bruxelles, Gosselies, Belgium.; 2 Cancer and Inflammation Program, Center for Cancer Research, National Cancer Institute, NIH, Bethesda, Maryland, USA.; 3 Signal Transduction Laboratory and; 4 Integrative Bioinformatics Support Group, National Institute of Environmental Health Sciences, Research Triangle Park, North Carolina, USA.; 5 Laboratoire de Biologie Moléculaire du Gène, ULB Center for Research in Immunology, Université Libre de Bruxelles, Gosselies, Belgium.; 6 Laboratory of Stem Cells and Cancer, WELBIO, and ULB Cancer Research Center, Université Libre de Bruxelles, Brussels, Belgium.; 7 Department of Biochemistry and Molecular Biology, Mayo Clinic, Rochester, New York, USA.; 8 Departments of Medicine and Biochemistry, Duke University Medical Center, Durham, North Carolina, USA.

**Keywords:** Inflammation, Oncology, Cytokines, Mouse models, Skin cancer

## Abstract

Cancer is caused primarily by genomic alterations resulting in deregulation of gene regulatory circuits in key growth, apoptosis, or DNA repair pathways. Multiple genes associated with the initiation and development of tumors are also regulated at the level of mRNA decay, through the recruitment of RNA-binding proteins to AU-rich elements (AREs) located in their 3′-untranslated regions. One of these ARE-binding proteins, tristetraprolin (TTP; encoded by *Zfp36*), is consistently dysregulated in many human malignancies. Herein, using regulated overexpression or conditional ablation in the context of cutaneous chemical carcinogenesis, we show that TTP represents a critical regulator of skin tumorigenesis. We provide evidence that TTP controlled both tumor-associated inflammation and key oncogenic pathways in neoplastic epidermal cells. We identify *Areg* as a direct target of TTP in keratinocytes and show that EGFR signaling potentially contributed to exacerbated tumor formation. Finally, single-cell RNA-Seq analysis indicated that *ZFP36* was downregulated in human malignant keratinocytes. We conclude that TTP expression by epidermal cells played a major role in the control of skin tumorigenesis.

## Graphical abstract

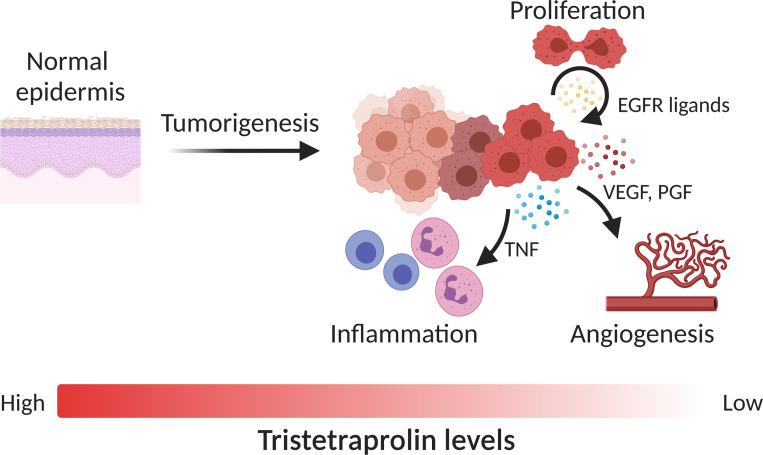

## Introduction

Tumors arise when gene regulatory circuits in key growth, apoptosis, or DNA repair pathways are dysregulated. Oncogenic events, such as mutations in protooncogenes, can radically affect cell physiology through downstream effects on gene expression, for example, by aberrant activation of transcription factors. Gene expression pathways may also be affected by alterations in posttranscriptional mechanisms, i.e., by modulating mRNA stability and/or their translational potential. Up to 10% of mammalian mRNAs harbor AU-rich elements (AREs) in their 3′-untranslated regions, allowing the recruitment of RNA-binding proteins that control their turnover and subcellular localization. One of these ARE-binding proteins is tristetraprolin (TTP; encoded by *ZFP36*). TTP targets these ARE-containing mRNAs for degradation by recruitment of deadenylase complexes. Decreased expression or function of TTP is consistently observed in many human malignancies, and multiple types of evidence now indicate that deregulation of ARE-mediated mRNA decay could represent a cardinal feature of tumor biology (1). For example, in breast tumor cell lines, miR-29a can regulate TTP expression, directly affecting epithelial polarity and metastasis (2). In hepatocellular carcinoma cell lines, hypermethylation of the *Zfp36* promoter favors tumor growth (3), whereas in glioma cell lines, hyperphosphorylated forms of TTP predominate, increasing VEGF and IL8 mRNA stability (4). Interestingly, *ZFP36* polymorphisms are associated with poor prognosis in patients with breast cancer (5). Myc oncoprotein directly suppresses TTP expression, leading to aberrant overexpression of ARE-containing mRNAs (6). Furthermore, oncogenic Ras signaling was shown to modulate TTP activity, leading to increased PD-L1 expression, indicating that this pathway may negatively affect antitumoral immune responses (7).

Importantly, TTP plays a major role in the control of inflammation, which represents a cardinal feature of tumor development. In macrophages and DCs, TTP controls the production of key inflammatory cytokines, such as TNF, IL-1β, IL-6, and IL-23 (8–10). Furthermore, in keratinocytes, TTP contributes to skin immune homeostasis by regulating TNF production (11). In line with these observations, TTP‑deficient mice spontaneously develop a complex TNF-dependent and IL-23–dependent inflammatory syndrome characterized by cachexia, progressive dermatitis, arthritis, and myeloid hyperplasia (9, 12). Herein, using a knockin model of regulated TTP overexpression (13) and a conditional TTP-deficient model (11) in the context of cutaneous chemical carcinogenesis, we show that TTP represents a critical regulator of skin tumorigenesis. We further provide evidence that TTP controls both tumor-associated inflammation and key oncogenic pathways in neoplastic epidermal cells.

## Results

### Regulated overexpression of endogenous TTP protected mice from cutaneous chemical carcinogenesis.

**Figure 1:**
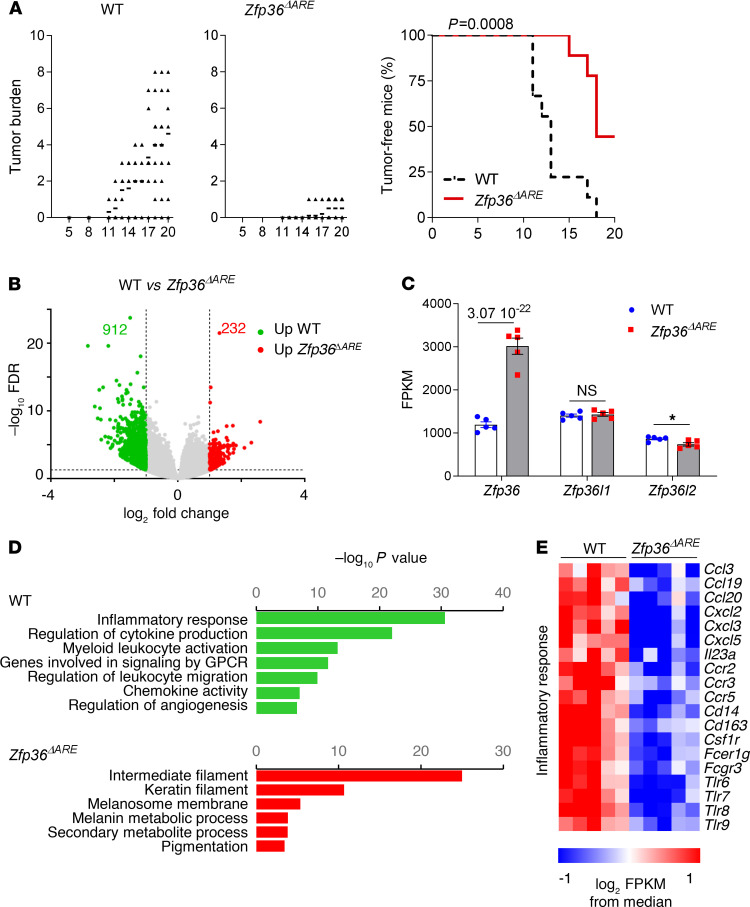
Regulated overexpression of endogenous TTP protects mice from cutaneous chemical carcinogenesis. *Zfp36^ΔARE^* mice and their controls (WT) were treated on shaved back skin with DMBA/TPA. They were monitored for 20 weeks at weekly intervals for tumor development. (**A**) Average tumor burdens and Kaplan-Meier curves describing tumor**-**free mice are shown for both groups (*n* = 9, representative of 2 experiments). (**B**) RNA-Seq analysis on adjacent, treated but nontumoral whole-thickness skin samples from both groups (*n* = 5). Differentially expressed genes are shown in the volcano plot in red if upregulated in *Zfp36^ΔARE^* skin (232 genes) or in green in WT skin (912 genes) among all 1144 genes that met the fold change (fold change > 2) and significance criteria (FDR < 0.05) (shown in gray). (**C**) *Zfp36* and other TTP family member mRNA expression in WT and *Zfp36^ΔARE^* mice, based on the RNA-Seq data from nontumoral adjacent skin samples. (**D**) Gene set enrichment analysis of the most significantly enriched pathways in *Zfp36^ΔARE^* (red) or in WT (green) samples. (**E**) Heatmap of expression levels of inflammatory response genes significantly increased or decreased in treated *Zfp36^ΔARE^* or WT skin. Statistical analysis (*P < 0.05) was assessed by Mantel-Cox log rank test indicating differences between all groups (*P* = 0.0008) (**A**) and using DeSeq2 (FDR = 3.07 10^-22^) (**C**). TTP, tristetraprolin; DMBA, 7,12-dimethylbenz[a]anthracene; TPA, 12-0-tetradecanoylphorbol-13-acetate.

Multiple lines of evidence support a major role for dysregulation of mRNA decay in cancer. However, most of the direct evidence for a role of TTP in cancer biology stems from studies using in vitro or transplantable tumor cell lines. It is clear that these models do not recapitulate the complex interactions between tumor, stromal, and immune cells. To explore the role of TTP in early phases of tumor development, we used the classic 2-step skin chemical carcinogenesis model in *Zfp36*^ΔARE^ mice. In these mice, a 136-base instability motif in the 3′-UTR of TTP mRNA was deleted in the endogenous genetic locus, leading to enhanced, systemic TTP protein expression and decreased susceptibility to several models of inflammatory disease (13). Consistent with previous reports (14), upon treatment with 7,12-dimethylbenz[a]anthracene (DMBA) followed by biweekly 12-0-tetradecanoylphorbol-13-acetate (TPA) application over the course of 10–18 weeks, all control mice developed multiple papillomas (Figure 1A). The tumor burden was strikingly reduced and delayed in the *Zfp36*^ΔARE^ mice, with half of the animals remaining tumor free after 20 weeks of treatment.

Upon termination of the experiment, we performed RNA-Seq on treated whole skin adjacent to papillomas (adjacent skin) from both groups. We observed 1144 statistically differentially expressed genes (DEGs; 232 and 912 upregulated and downregulated genes in *Zfp36*^ΔARE^ mice, respectively, with a fold change greater than 2 and FDR less than 0.05) (Figure 1B). As expected, *Zfp36* mRNA expression was increased by approximately 3-fold in the skin of *Zfp36*^ΔARE^ mice (Figure 1C). Furthermore, expression of its paralogs, *Zfp36l1* and *Zfp36l2*, was largely equivalent in both groups, whereas the placenta- and yolk sac–specific paralog *Zfp36l3* was undetectable. We observed a strong enrichment for innate immunity, myeloid cells, and inflammation-related pathways among genes that were downregulated in the *Zfp36*^ΔARE^ samples, indicating that TTP overexpression restricts inflammation in these chronically stimulated skin samples (Figure 1D). This is consistent with previous results showing that *Zfp36*^ΔARE^ mice are protected from imiquimod-induced dermatitis (13). In addition to known TTP targets (e.g., *Cxcl2* or *Il23a*), many genes expressed by myeloid cells (*Cd163, Cd14, Fcer1g, Csfr1, Tlrs*) were found to be decreased, suggesting decreased recruitment and activation of innate immune cells (Figure 1E). Taken together, these data indicate that increased systemic TTP expression from its endogenous locus protects mice from inflammation-induced skin carcinogenesis.

### Expression of TTP by epidermal cells rather than myeloid cells played a major role in the control of skin carcinogenesis.

**Figure 2:**
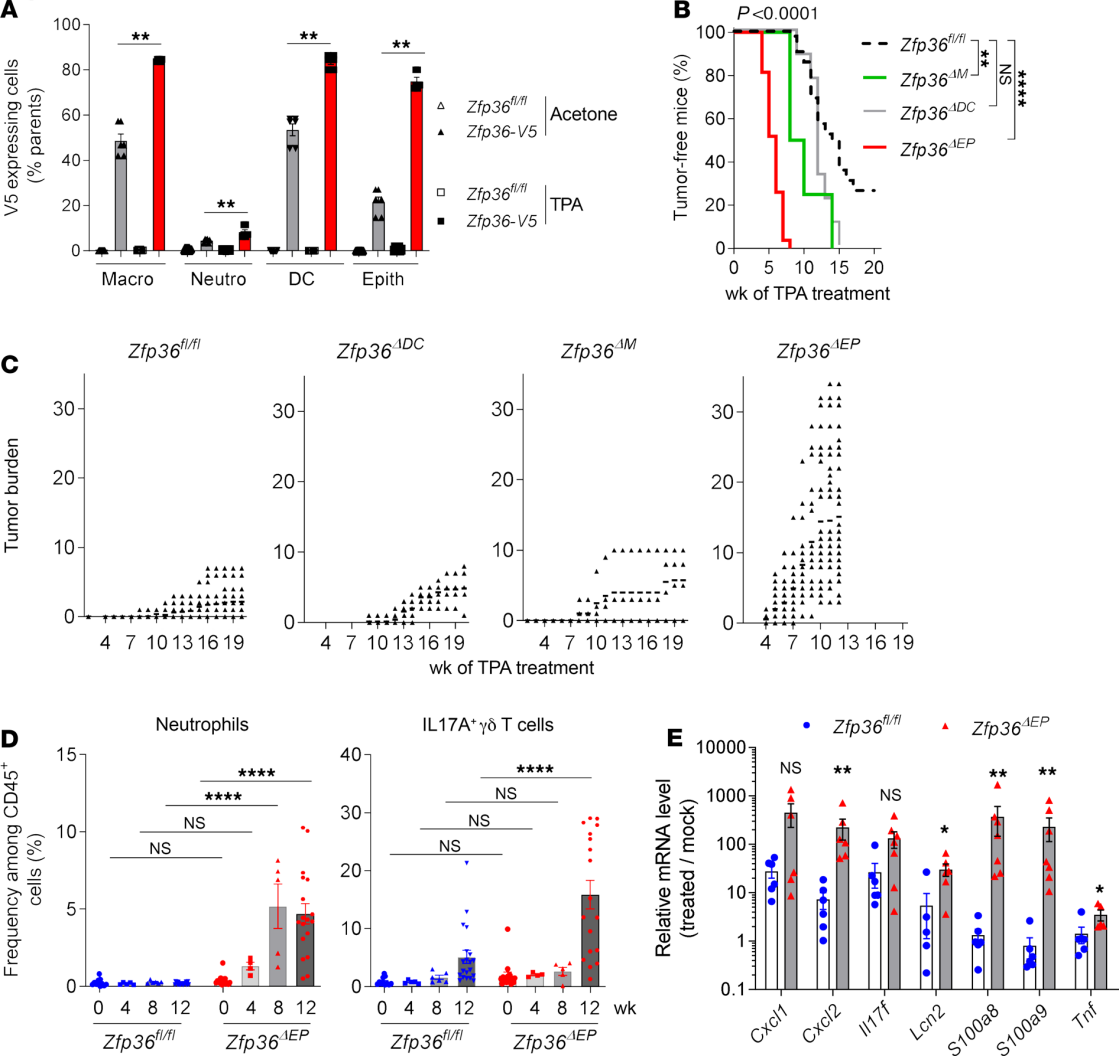
TTP expressed by keratinocytes controls skin tumorigenesis. *Zfp36-V5* and *Zfp36^fl/fl^* mice were treated for 3 days with TPA or acetone on shaved back skin (**A**). *Zfp36^ΔDC^*, *Zfp36^ΔM^*, *Zfp36^ΔEP^* mice and their littermate controls (*Zfp36^fl/fl^*) were topically treated on back skin with DMBA/TPA for 12 to 20 weeks and monitored every week for tumor formation. The experiment was stopped when the tumors reached > 10 mM (**B–E**). (**A**) Ratio of V5-expressing skin cells in each condition analyzed by flow cytometry: macrophages, neutrophils, DCs, and epithelial cells (keratinocytes). Data are shown as percentage of the initial population (mean ± SEM, *n* = 6, representative of 2 experiments). (**B**) Kaplan-Meier plot of DMBA/TPA-treated mice depicting papilloma-free state after TPA promotion (*n* = 4–42, pool of 6 experiments). (**C**) Tumor burdens of *Zfp36^fl/fl^, Zfp36^ΔEP^*, *Zfp36^ΔM^, and Zfp36^ΔDC^* back skins. (**D**) Kinetics of cell infiltration in total back skin from *Zfp36^ΔEP^* mice: neutrophils and IL-17A–producing γδT cells among CD45^+^ cells are shown by intracellular protein staining. Results are given as mean ± SEM (*n* = 4–27, pool of 4 experiments). Gating strategy for flow cytometry is presented in Supplemental Figures 1 and 2. (**E**) Skin samples, including tumors, from *Zfp36^ΔEP^* mice and their littermates were collected for analysis of transcript levels by qPCR at 12 weeks of DMBA/TPA treatment. Results are expressed as relative to the *Zfp36^fl/fl^* mock group, which was arbitrarily set to 1 (mean ± SEM, *n* = 7). Statistical significance (*P < 0.05, **P < 0.01, ****P < 0.0001) was assessed by 2-tailed Mann-Whitney test (**A** and **E**), by Mantel-Cox log rank test and pairwise comparisons indicating differences between deficient mice compared with their controls (**B**) or by the 1-way ANOVA test with Bonferroni’s correction compared with the *Zfp36^fl/fl^* group (**D**). TTP, tristetraprolin; DMBA, 7,12-dimethylbenz[a]anthracene; TPA, 12-0-tetradecanoylphorbol-13-acetate.

To define in which cell types TTP was active in preventing skin carcinogenesis, we then assessed TTP protein expression in cells isolated from the skin after short-term treatment with TPA. Unfortunately, most available anti-TTP antibodies cannot be reliably used for FACS staining. To circumvent this technical problem, we used a *Zfp36-V5* epitope–tagged knockin mouse generated by CRISPR/Cas9-mediated genome editing (15). We observed consistent TTP-V5 staining in myeloid cells from acetone-treated mice that was further increased upon TPA treatment. The highest expression levels were observed in macrophages and DC subsets. In parallel, we assessed TTP-V5 expression in keratinocytes. We detected low levels after acetone treatment, but markedly increased expression after TPA treatment, reaching levels comparable with those observed in macrophages or DCs from TPA-treated skin (Figure 2A). Based on this result, we decided to evaluate the contribution of TTP expressed in these different cell types. For this purpose, we deleted *Zfp36* in myeloid cells (LysMCre-*Zfp36^fl/fl^*; *Zfp36*^ΔM^** mice, targeting macrophages, neutrophils, and monocytes), DCs (CD11cCre-*Zfp36^fl/fl^;* *Zfp36*^ΔDC^** mice), or epidermal cells (K14Cre-*Zfp36*^fl/fl^; *Zfp36*^ΔEP^** mice) and subjected these mice to DMBA/TPA treatment. We observed a modest increase in tumor burden and size in *Zfp36*^ΔDC^** and *Zfp36*^ΔM^** mice compared with that in control *Zfp36^fl/fl^* mice. In sharp contrast, we found that TTP deletion in epidermal cells (*Zfp36*^ΔEP^** mice) resulted in a dramatic increase in tumorigenesis. Control mice needed at least 8 weeks of TPA treatment for the development of the first papillomas, whereas *Zfp36*^ΔEP^** mice displayed more rapid tumor formation (in less than 4 weeks), with major increases in tumor burden, size, and progression to carcinoma (Figure 2, B and C). In addition, the development of these tumors was accompanied by a gradual recruitment of neutrophils and, to a lesser extent, of IL-17A–producing cells (mostly dermal γδ T cells) in the skin, indicating an essential role of TTP within keratinocytes in the control of inflammation-related carcinogenesis (Figure 2D). This was associated with upregulated expression of genes encoding cytokines (*Tnf* or *Il17f*), chemokines (*Cxcl1*, *Cxcl2*), and antimicrobial peptides (*Lcn2*, *S100a8*, *S100a9*) (Figure 2E). Taken together, these results highlight the key role of TTP within epidermal cells in the control of chronic inflammation and tumorigenesis.

### Heightened tumor development upon epidermal-specific TTP ablation was not driven by dysregulated TNF production.

**Figure 3:**
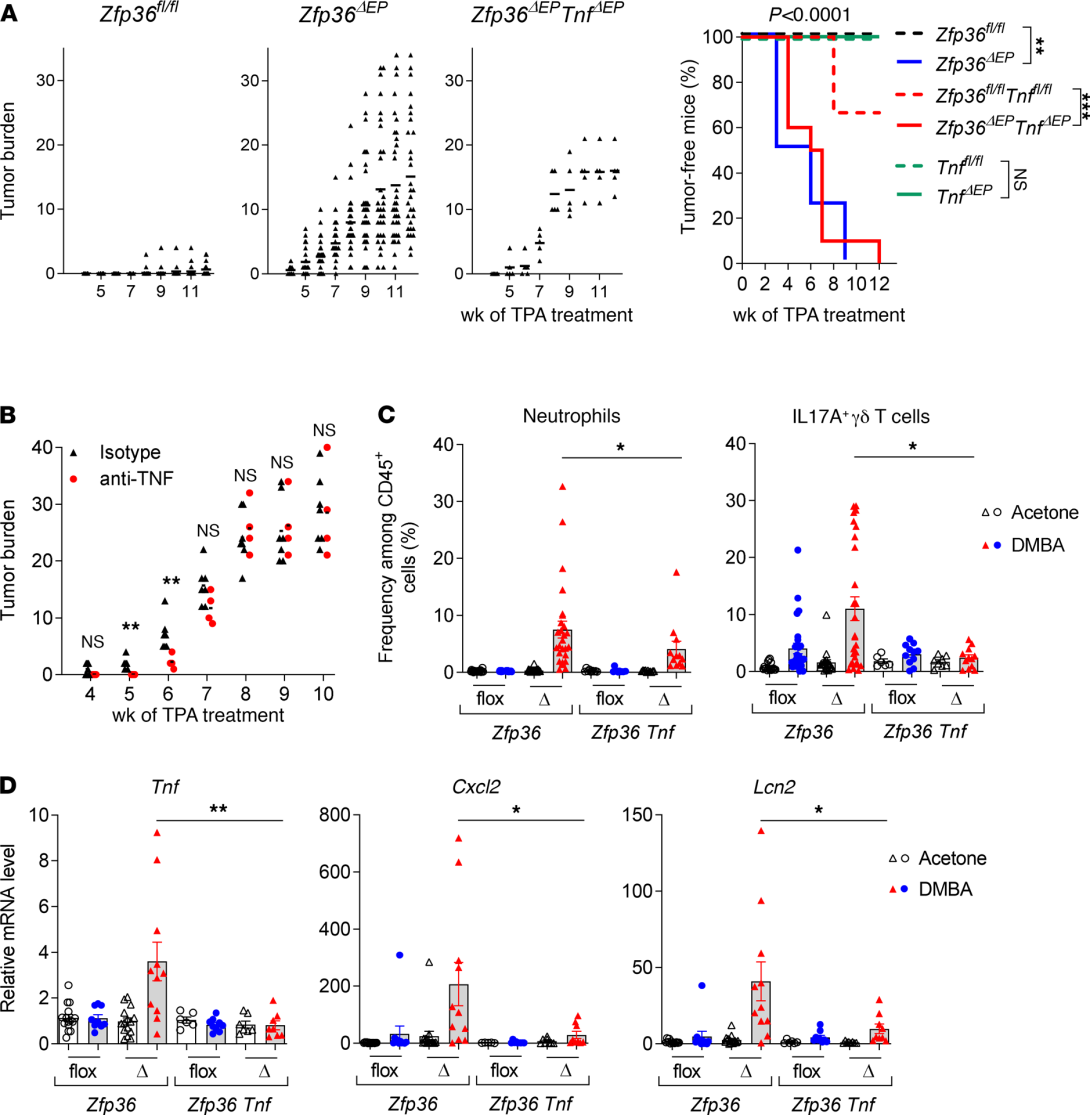
Dysregulated production of TNF in the absence of TTP in keratinocytes does not critically contribute to the sensitivity of *Zfp36^ΔEP^* mice to tumor development. Tumors from *Zfp36^ΔEP^Tnf^ΔEP^* (red), *Tnf^ΔEP^* (green), *Zfp36^ΔEP^* (blue) mice and their littermates (dotted lines) were monitored during 12**–**20 weeks after DMBA/TPA treatment. (**A**) Incidence and size of tumors of all groups. Kaplan-Meier curves showing tumor-free mice during the experiment (*n* = 4–11, pool of 2 experiments). (**B**) DMBA/TPA-treated *Zfp36^ΔEP^* mice received anti–TNF-α or control isotype (*n* = 4–5, representative of one experiment). (**C**) Cell recruitment in total back skin was analyzed by flow cytometry (mean ± SEM, *n* = 4–11, pool of 2 experiments). Gating strategy for flow cytometry is presented in Supplemental Figure 2. (**D**) Total back skin was collected for gene expression analysis by qPCR. Deficient mice (Δ) and their littermates (flox) are shown for both groups. Levels of *Tnf*, *Cxcl2*, and *Lcn2* mRNAs were measured for each condition. Values from mock skin of corresponding controls were normalized against *Actb* and arbitrarily set at 1 (mean ± SEM, *n* = 6–11). Statistical significance (*P < 0.05, **P < 0.01, ***P < 0.001) was assessed by Mantel-Cox log rank and pairwise comparisons (**A**), by 2-tailed Mann-Whitney test (**B–D**). TTP, tristetraprolin; DMBA, 7,12-dimethylbenz[a]anthracene; TPA, 12-0-tetradecanoylphorbol-13-acetate.

TNF, whose mRNA is the best-characterized direct target of TTP (8), is involved in tumor promotion and progression in a wide range of genetic, chemically induced, and transplantable mouse models of cancer, including skin carcinogenesis (16). Because we observed dysregulated *Tnf* expression in the skin of DMBA/TPA-treated *Zfp36*^ΔEP^** mice, we generated mice with specific ablations of both TNF and TTP in keratinocytes (K14Cre*Tnf^fl/fl^Zfp36^fl/fl^*; *Zfp36*^ΔEP^*Tnf*^ΔEP^** mice). Using this approach, we previously demonstrated that the exacerbated imiquimod-induced skin inflammation seen in the *Zfp36*^ΔEP^** mice was strongly dependent on the capacity of keratinocytes to produce TNF (11). In sharp contrast, these mice still displayed extreme sensitivity to DMBA/TPA-induced tumor formation, indicating that, in this case, dysregulated production of TNF in the absence of TTP in epidermal cells is not the primary driver (Figure 3A). In addition, we performed similar experiments using neutralizing anti-TNF antibodies in *Zfp36*^ΔEP^** mice. Although we observed a decrease in tumor burden at early time points, the numbers and sizes of the tumors were found to be comparable in isotype- and anti-TNF–treated *Zfp36*^ΔEP^** mice at later stages (Figure 3B). Nonetheless, we observed that accumulation of neutrophils and IL-17A–producing γδ T cells was dependent on epidermal cell–derived TNF (Figure 3C). Furthermore, expression of inflammatory genes (*Tnf* but also *Cxcl2* or *Lcn2*) was also strongly reduced in these conditions compared with *Zfp36*^ΔEP^** mice (Figure 3D). Taken together, these results suggest that a heightened inflammatory state was not central to increased tumorigenesis in these mice.

### TTP regulated multiple key oncogenic pathways in neoplastic epidermal cells.

**Figure 4:**
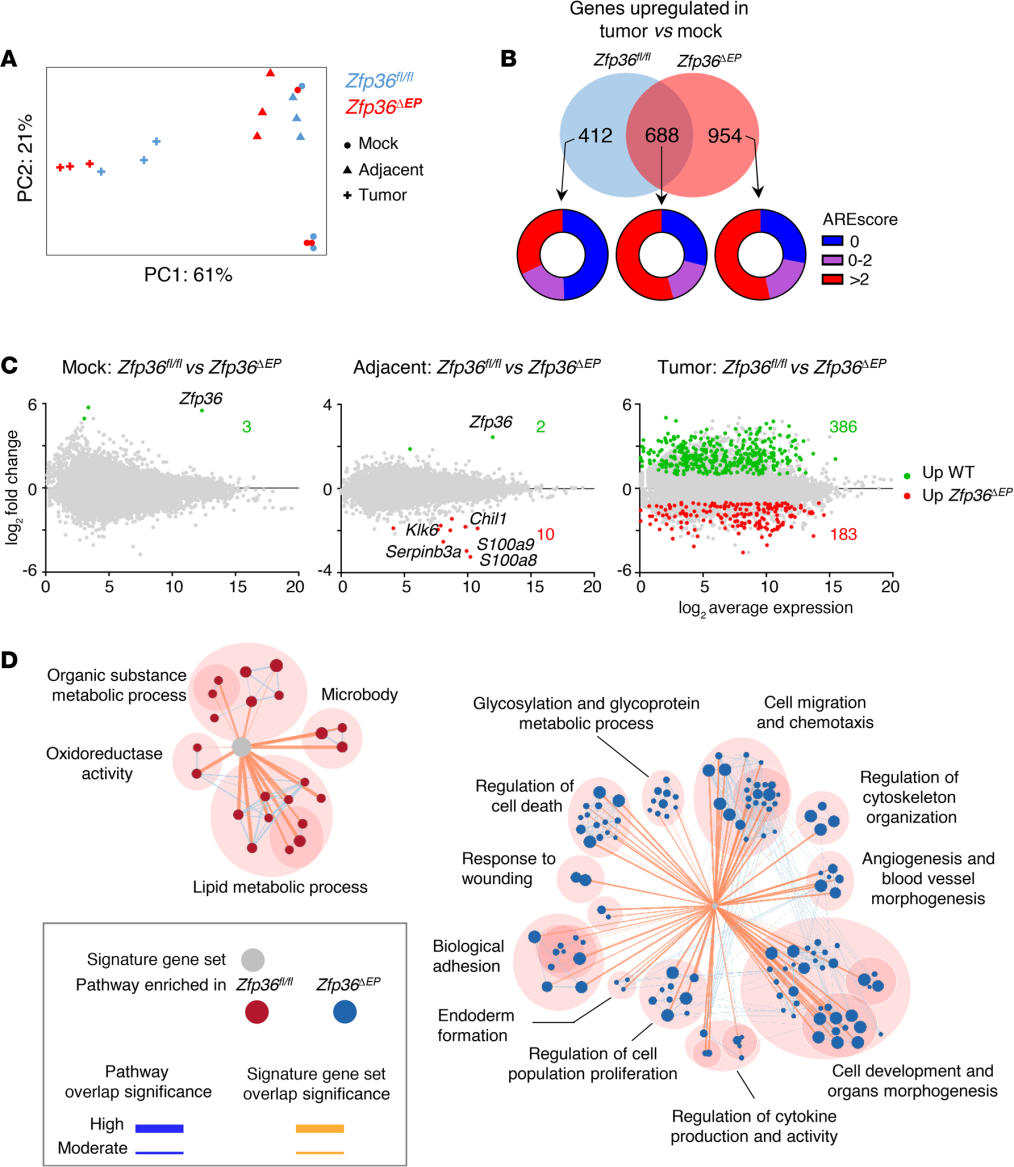
TTP shapes the transcriptome of epidermal cells upon neoplastic transformation. Epithelial cells from tumors and adjacent-treated and mock-treated skin of *Zfp36^ΔEP^* (red) mice and their littermates (blue) were isolated by flow cytometry after DMBA/TPA treatment for RNA-Seq analysis (*n* = 3). (**A**) PCA analysis showing segregation of samples. (**B**) Number of genes upregulated in tumors compared with mock skin samples. ARE score frequencies of dysregulated genes are shown for the indicated categories. (**C**) M-A plots indicating upregulated (red) and downregulated (green) genes in *Zfp36^ΔEP^* for cells isolated from mock-treated skin, adjacent-treated skin, and tumors. (**D**) Differentially expressed genes analysis of *Zfp36^fl/fl^* (WT, red) and *Zfp36^ΔEP^* (blue) tumor cells. Signature gene sets represent upregulated genes in WT tumors (386 genes, left part) and *Zfp36^ΔEP^* (183 genes, right part). Overlaps between pathways are indicated in blue lines, and the overlaps between signature gene sets is indicated in orange. The thicker the line is, the more enriched genes are present between the 2 pathways. TTP, tristetraprolin; DMBA, 7,12-dimethylbenz[a]anthracene; TPA, 12-0-tetradecanoylphorbol-13-acetate.

Although keratinocyte-derived TNF plays a dominant role in other disease settings, this does not seem to be the case in the current context of carcinogen-induced skin tumor formation. TTP can potentially control the mRNA stability of multiple inflammatory and oncogenic molecules. With the aim of defining the global impact of TTP in epidermal cells, we performed transcriptomic analysis. After induction of tumors in *Zfp36^fl/fl^* and *Zfp36*^ΔEP^** mice, we sorted EpCAM^+^CD45^-^CD140a^-^CD31^-^ epidermal cells from papillomas at the same stage of development (as depicted in Supplemental Figure 3; supplemental material available online with this article; https://doi.org/10.1172/jci.insight.140669DS1). As comparators, we also sorted epidermal cells from adjacent DMBA/TPA-treated skin and from mock-treated animals. This allowed us to identify DEGs associated with chronic stimulation and/or specific to oncogenic transformation. Principal component analysis (PCA) showed a clear segregation between neoplastic and adjacent/mock-treated samples (Figure 4A). Importantly, TTP-deficient and *Zfp36^fl/fl^* tumor cells tended to form separate clusters. Next, we performed pairwise comparisons. When comparing mock-treated versus tumor conditions, we identified 1100 and 1642 significantly upregulated genes in *Zfp36^fl/fl^* or *Zfp36*^ΔEP^** groups, respectively. We used the ARE score algorithm to identify potential direct targets of TTP among dysregulated genes (Figure 4B) (17). When considering the genes that were upregulated in the *Zfp36*^ΔEP^** group, we observed a higher frequency of transcripts with an ARE score greater than 2, compared with genes that were specifically increased in *Zfp36^fl/fl^* cells only. In line with the PCA data, we identified very few DEGs between *Zfp36^fl/fl^* and *Zfp36*^ΔEP^** groups in mock-treated conditions (3 genes) and DMBA/TPA nontumoral skin (12 genes). Nevertheless, in this latter condition, we identified important inflammatory genes such as *Chil1*, *S100a8*, and *S100a9*. Of note, expression of *Klk6*, the gene encoding a proinflammatory peptidase that promotes skin tumor formation and progression (18), was increased in TTP-deficient cells (Figure 4C). This was also the case for *Serpinb3a*, the gene encoding one of the squamous cell carcinoma antigen 1–related molecules, commonly used as prognostic biomarkers in cancer patients (19).

**Figure 5:**
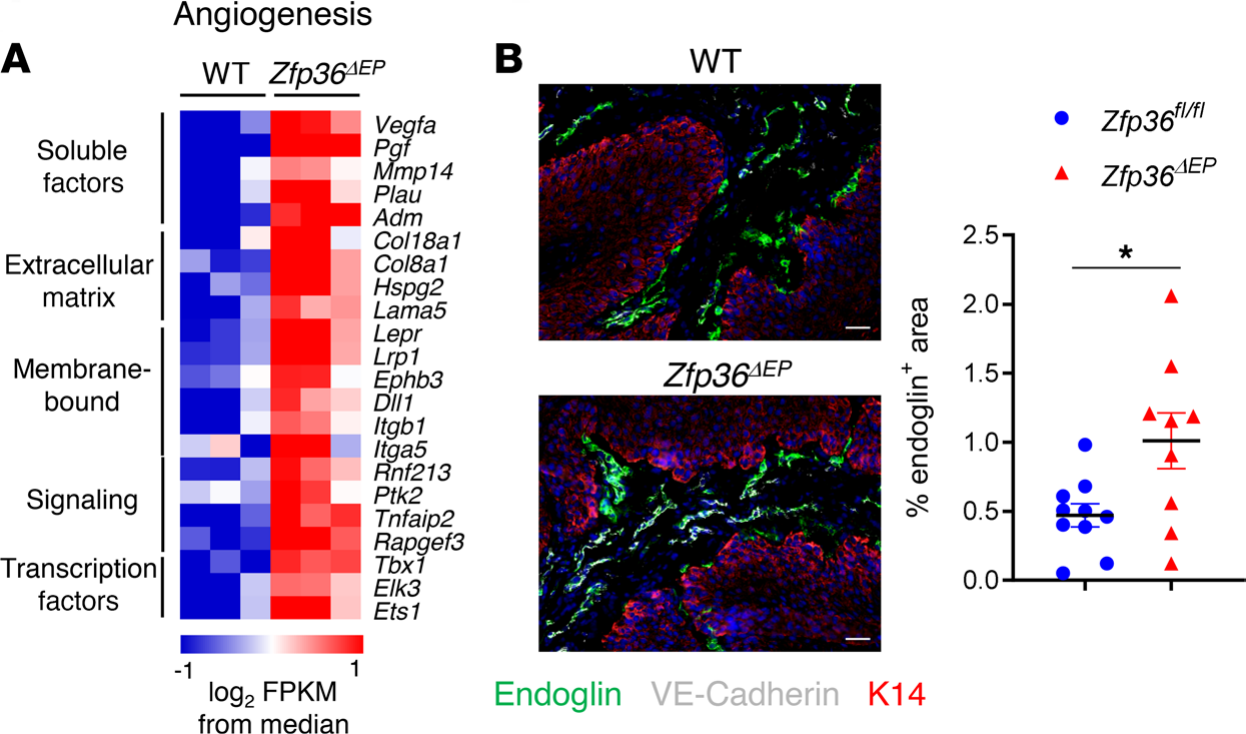
Increased neovascularization of *Zfp36^ΔEP^* papillomas. (**A**) Heatmap of expression levels of angiogenesis-related genes significantly increased in *Zfp36*^ΔEP^ tumor cells compared with their WT counterparts. (**B**) Sections of papillomas (3-mM diameter) from *Zfp36^ΔEP^* and *Zfp36^fl/fl^* mice after 12 weeks of treatment. Cryosections were stained with endoglin (green), VE-Cadherin (gray), Keratin14 (red), and nuclei (blue). The relative surface of endoglin-positive staining in papilloma sections was measured and graphed (mean ± SEM, *n* = 9–10). Statistical significance (*P < 0.05) was assessed by 2-tailed Mann-Whitney test.

For epidermal cells isolated from papillomas, using the same criteria, we identified 569 DEGs between *Zfp36^fl/fl^* and *Zfp36*^ΔEP^** groups. Apart from lipid metabolism, gene set enrichment analysis revealed few relevant pathways associated with genes that were downregulated in TTP-deficient cells. In contrast, important pathways were enriched for genes that were upregulated upon TTP ablation. As expected, we observed increased expression of genes involved in cytokine expression and activity, such as *Tnf*, *Il1b*, and *Cxcl1*. We also noted dysregulation of key oncogenic processes including cell death, migration, proliferation, and angiogenesis (Figure 4D). Among angiogenesis-related genes that were significantly upregulated in *Zfp36*^ΔEP^** tumor cells, we identified key soluble factors such as *Vegfa* but also multiple genes involved in interactions with stromal cells (Figure 5A). To evaluate angiogenesis and neovascularization of *Zfp36^fl/fl^* and *Zfp36*^ΔEP^** papillomas, we performed immunofluorescent staining for VE-Cadherin (an endothelial marker) and endoglin, a transmembrane glycoprotein expressed on activated vascular endothelial cells (20). We observed increased density of endoglin^+^ cells in *Zfp36*^ΔEP^** papillomas compared with *Zfp36^fl/fl^* samples, indicating that the absence of TTP in tumor cells promoted angiogenesis (Figure 5B). We conclude that TTP shapes the transcriptome of epidermal cells upon neoplastic transformation by controlling the expression of mRNAs associated with key oncogenic pathways such as neovascularization.

### TTP destabilized Areg mRNA in neoplastic epidermal cells.

**Figure 6:**
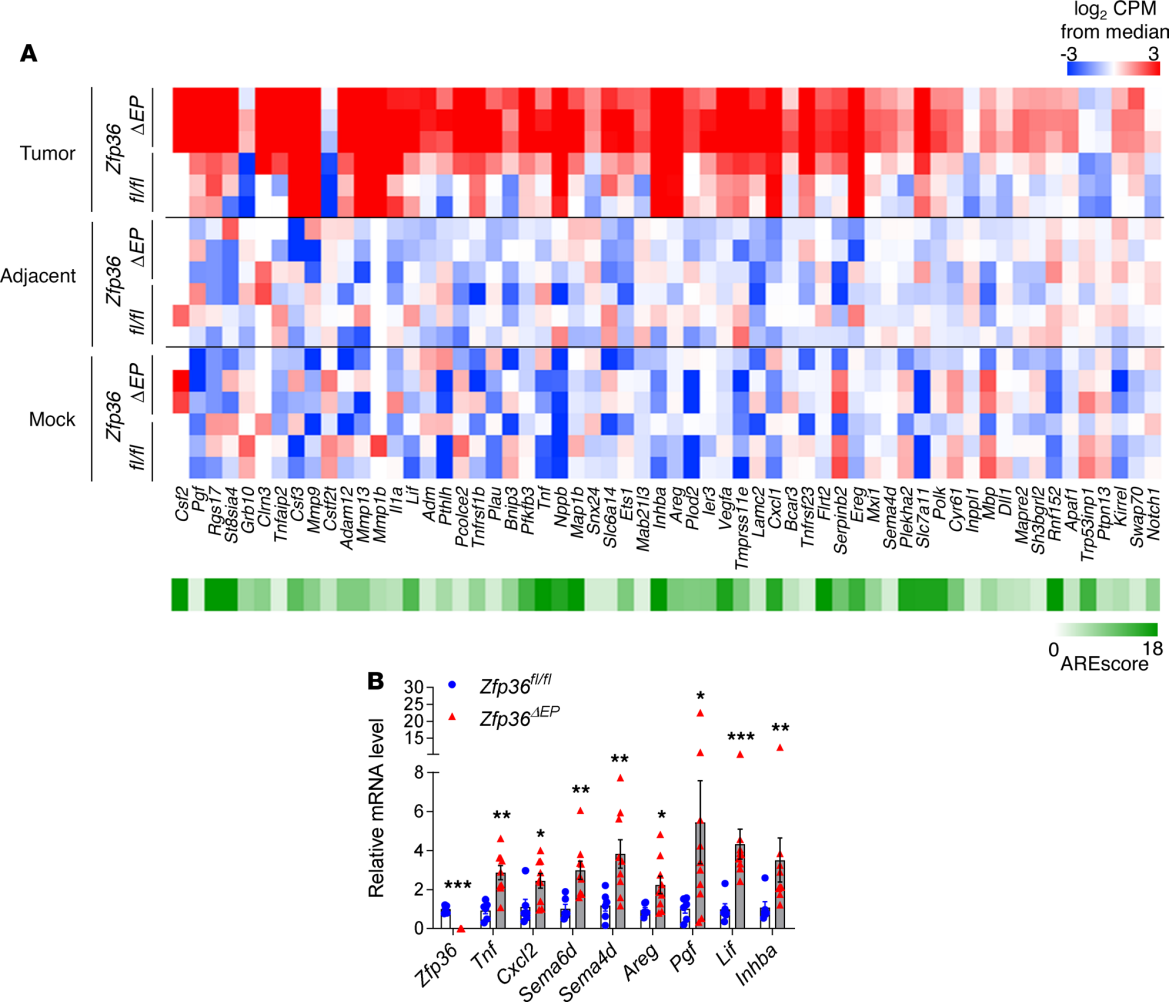
Potential direct targets of TTP in neoplastic epidermal cells. Epithelial cells from tumors and adjacent-treated and mock-treated skin of *Zfp36^ΔEP^* mice and their littermates were isolated for RNA-Seq analysis (*n* = 3). (**A**) Heatmap of expression levels of transcripts significantly increased or decreased for indicated groups and ARE score of tumor-specific transcripts from *Zfp36^ΔEP^* and *Zfp36^fl/fl^* samples is shown when > 2. (**B**) mRNA levels of sorted papilloma cells analyzed by qPCR. Levels are normalized against *Actb* and expressed relative to values from the *Zfp36^fl/fl^* mice, arbitrarily set to 1 (mean ± SEM, *n* = 7–11, pool of 3 experiments). Statistical significance (*P < 0.05, **P < 0.01, ***P < 0.001) was assessed by 2-tailed Mann-Whitney test. TTP, tristetraprolin; TPA; ARE, AU-rich element.

To identify direct potential targets of TTP in tumor cells, we looked at tumor-specific transcripts with an ARE score greater than 2 that were significantly upregulated (FC > 2) in the *Zfp36*^ΔEP^** group compared with their control counterparts (Figure 6A). For selected genes, we validated our results by RT-qPCR on sorted epidermal cells from independent papilloma samples (Figure 6B). Several of these 60 genes were previously shown to be directly regulated by TTP in other cell types. These include genes encoding important cytokines, growth factors, chemokines, or enzymes such as *Tnf* (21), *Csf2* (22), *Il1a* (10)*, Lif* (23), *Vegfa* (4), *Cxcl1* (24), or *Mmp9* (25). We also identified several relevant mediators that, to the best of our knowledge, have not been reported as TTP targets. For example, we observed increased expression of *Pgf*, encoding placental growth factor, a VEGF homolog that plays an important role in ischemic, inflammatory, and malignant diseases (26). Along the same lines, we also highlight activinβA (*Inhba*) because this member of the TGF-β superfamily was shown to increase malignancy and metastatic spread of skin tumors (27); semaphorins (*Sema6d*, *Sema4d*), which can shape the tumor microenvironment (28); and ligands of the EGF receptor (*Areg*, *Ereg*). EGFR signaling in epidermal cells plays an essential role downstream of the oncogenic RAS pathway (29). We therefore assessed whether *Areg* represents a bona fide TTP target.

**Figure 7:**
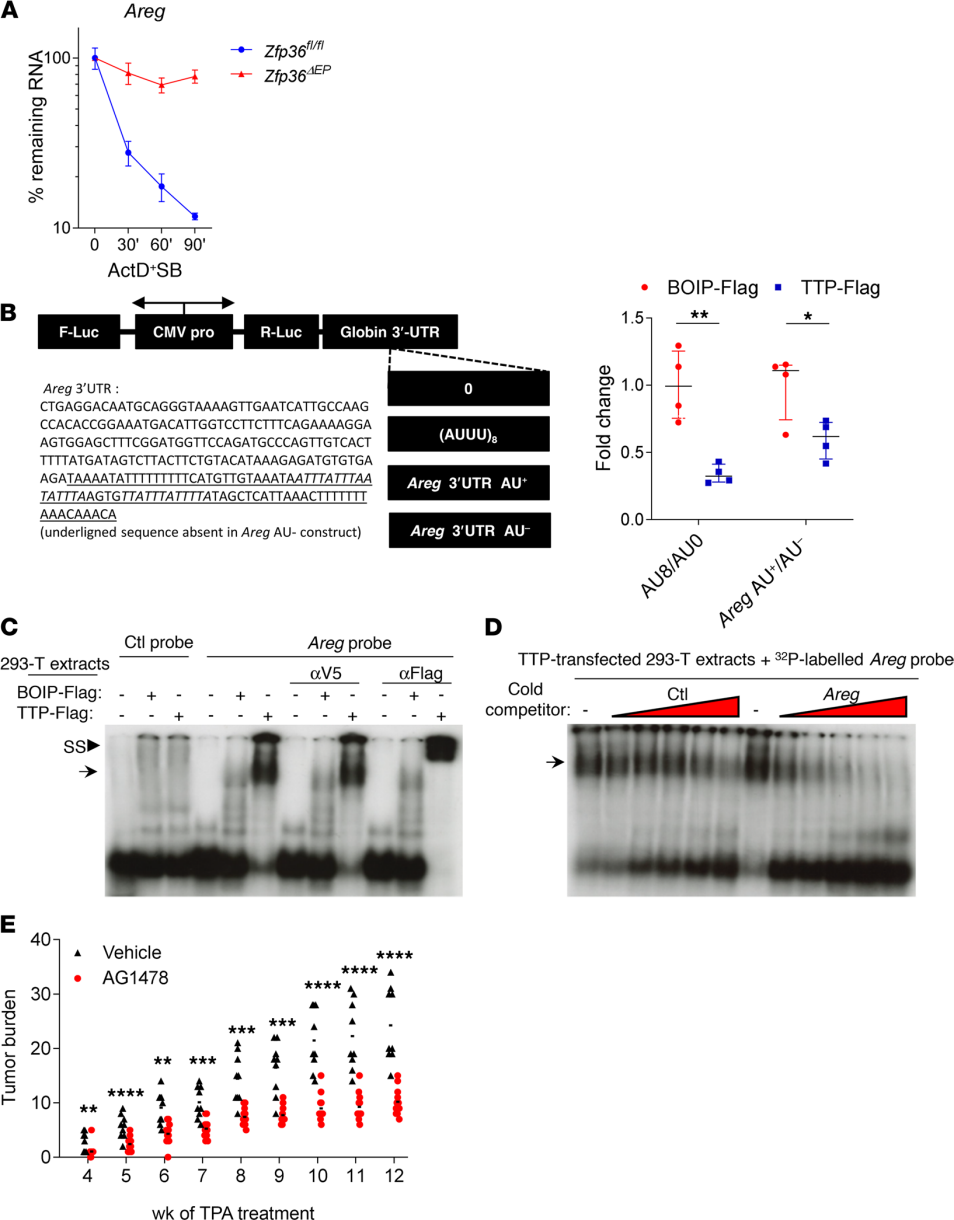
TTP destabilizes *Areg* mRNA in neoplastic epidermal cells. (**A**) Primary keratinocytes isolated from *Zfp36^fl/fl^* or *Zfp36^ΔEP^* newborns were stimulated with TPA (100 ng/mL) for 2 hours prior to actinomycin D (10 μg/μl) and SB202190 (1 μM) treatment for the indicated time. Half-life of *Areg* mRNA mRNA was quantified by qPCR and normalized by mRNA levels at t_0_ (mean ± SEM, *n* = 3–4, representative out of 2 experiments). (**B**) Schematic representation of the dual-reporter plasmids generated to assess the role of the ARE elements: 3’UTR of *Areg* mRNA with (AU+) or without (AU-) ARE was inserted behind the sequence coding for *Renilla* luciferase (R-Luc). Fold change between the Rluc/Fluc ratio of AU+ and AU- luciferase signals from dual-reporter plasmids cotransfected with TTP-Flag (or BOIP-Flag as control) in HEK293T cells (mean ± SEM, representative out of 7 experiments). (**C**) Electrophoretic mobility supershift assay. Extracts from HEK293T cells transiently transfected with TTP-Flag or BOIP-Flag were incubated with a ^32^P-labeled probe of *Areg* ARE or a CTRL probe corresponding to pBS polylinker and no antibody, an α-V5, or an α-Flag antibody before migration.(**D**) ^32^P-labeled Areg probe mixed with increasing molar excess of unlabeled Areg probe or pBS control probe was used to performed EMSA competition experiment. (**C** and **D**) Results are representative of 3 independent experiments. (**E**) *Zfp36^ΔEP^* mice were treated with DMBA/TPA, and 45 minutes before each application of TPA, mice were treated with the EGFR inhibitor AG1478 (500 μg/mL) or vehicle. Mice were monitored for 12 weeks at weekly intervals for tumor development (*n* = 8–13, representative of 2 experiments). Statistical significance (*P < 0.05, **P < 0.01, ***P < 0.001, ****P < 0.0001) was assessed by 2-way ANOVA (**B**) and 2-tailed Mann-Whitney test (**E**). TTP, tristetraprolin; DMBA, 7,12-dimethylbenz[a]anthracene; TPA, 12-0-tetradecanoylphorbol-13-acetate; ARE, AU-rich element; pBS, pBluscript polylinker region.

We first performed in vitro experiments with primary *Zfp36^fl/fl^* and *Zfp36*^ΔEP^** keratinocytes. After a short incubation with TPA to induce TTP expression, we evaluated *Areg* mRNA half-life by treating the cells with actinomycin D and SB203580, the latter of which is used to abrogate the inhibitory action of p38 MAPK on TTP activation (10). As shown in Figure 7A, *Areg* mRNA stability was strongly increased in *Zfp36*-deficient keratinocytes. Next, to assess if the putative ARE sequences found in the 3′UTR of the mRNA coding for AREG were sufficient to promote mRNA destabilization by TTP, we used a bidirectional reporter system (30) in which we inserted either the full-length 3′UTR sequence of *Areg* or a truncated version lacking ARE motifs (Figure 7B). Plasmids containing a synthetic (AUUU)8 ARE motif (AU8) or no ARE (AU0) were used as controls. We evaluated the effect of TTP in cotransfection experiments in HEK293T cells. As expected, we observed a clear effect of TTP on the ratio between AU8 and AU0 reporter activities compared with a control plasmid (expressing BOIP, encoded by *Ccdc89*) (Figure 7B). Similar conclusions were reached for the construct containing *Areg* 3′UTR, with and without ARE motifs.

To test the capacity of TTP to directly bind *Areg* ARE, we performed an electrophoretic mobility supershift assay. We incubated the *Areg* ARE probe with extracts from TTP-Flag–expressing HEK293T cells. Addition of α-Flag antibody resulted in a supershift that was not observed with BOIP-Flag or with an anti-V5 antibody, demonstrating that TTP physically interacts with the *Areg* mRNA ARE in this in vitro setting (Figure 7C). We confirmed the specificity of this binding by showing competition with increasing concentrations of cold *Areg* ARE but not with a control probe (Figure 7D). Finally, to define whether EGFR signaling plays a dominant role in the exacerbated tumorigenesis displayed by *Zfp36*^ΔEP^** mice, we treated them before each TPA application with the EGFR inhibitor AG1478. As shown in Figure 7E, the number and size of the papillomas were greatly reduced upon treatment with this tyrosine kinase inhibitor. Taken together, these results indicate that TTP directly controlled *Areg* mRNA stability in keratinocytes, and that dysregulated EGFR signaling may contribute to exacerbated tumor formation in *Zfp36*^ΔEP^** mice.

### ZFP36 was downregulated in human squamous cell carcinoma.

**Figure 8:**
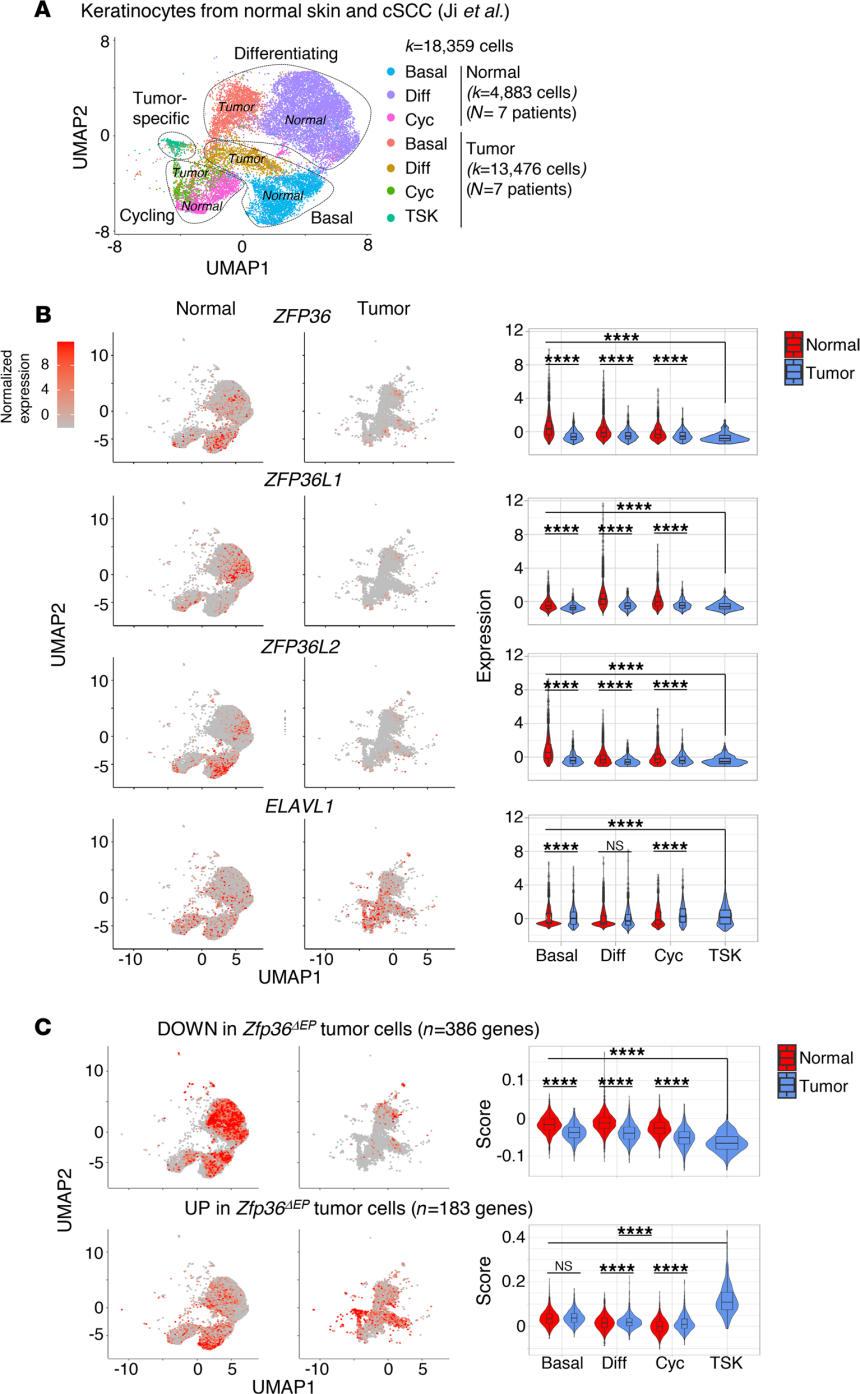
Downregulation of *ZFP36* expression in human tumor-specific keratinocytes. (**A**) Uniform manifold approximation and projection (UMAP) of 18,359 single-cell RNA-Seq keratinocytes recovered from the skin of 7 control individuals and from 7 patients with cutaneous squamous cell carcinoma (cSCC) labeled by subsets and origins. (**B**) Expression of the indicated genes was measured and visualized on UMAP after separating tumor from normal cells or by violin plots. (**C**) Enrichment scores of genes that were found to be downregulated (upper panel) or upregulated (lower panel) in *Zfp36^ΔEP^* tumor cells visualized on UMAP or by violin plots. Statistical significance (****P < 0.0001) was assessed by 1-way ANOVA with post hoc Tukey’s HSD test.

Because our mouse models revealed a clear role of TTP in skin carcinogenesis, we investigated whether our observations might translate to the clinic. TTP is expressed in multiple cell types in the tumor microenvironment. To specifically look at the role of TTP in epidermal cells, we analyzed a recently published single-cell RNA-Seq data set of 18,359 keratinocytes taken from the skin of 7 control individuals and from 7 patients with cutaneous squamous cell carcinoma (cSCC) (31). As shown in Figure 8A, 3 major subpopulations were defined in normal skin. Based on the expression of representative genes, these were defined as basal (*COL17A1*), cycling (*MKI67*), and differentiating (*KRT1*) keratinocytes. cSCC exhibited 4 subpopulations, 3 recapitulating these normal epidermal states, and a tumor-specific keratinocyte (TSK) population with no counterpart in normal skin. These TSKs were shown to express epithelial-mesenchymal transition markers and to reside within a fibrovascular niche at leading edges of the tumor (31). We evaluated expression of *ZFP36* in these 7 cell clusters (Figure 8B). We observed lower expression in each of the 3 tumor subpopulations compared with their normal counterparts. In addition, its expression was further reduced in TSK cells. We observed very similar patterns for its 2 paralogs, *ZFP36L1* and *ZFP36L2*. In sharp contrast, expression of *ELAVL1*, that codes for another ARE-binding protein with opposite functions, was higher in tumor cells and TSKs in particular. We evaluated the signature scores for the 386 and 183 genes that were downregulated or upregulated in TTP-deficient neoplastic epidermal cells (as defined in Figure 4C). Consistent with their lower expression of *ZFP36*, TSKs exhibited the lowest and highest expression of these hallmark gene signatures (Figure 8C). Globally, these results suggest that ARE-mediated mRNA decay was dysregulated in human cSCC.

## Discussion

We show here that TTP played a major role in the pathogenesis of skin carcinogenesis. When its regulated expression was increased throughout the body by genetically removing the ARE instability elements that are located in its own mRNA 3′UTR (*Zfp36*^ΔARE^** knockin mice), DMBA/TPA-induced tumor burden was greatly reduced. Conversely, TTP ablation specifically in epidermal cells led to extreme sensitivity to DMBA/TPA-induced tumor formation. Because TTP plays a major role in the control of inflammatory cytokine and chemokine expression, it was not surprising that both experimental situations were associated with important effects on tumor-associated inflammation. As previously demonstrated in the context of imiquimod-induced dermatitis (11), dysregulated production of TNF in the absence of TTP in epidermal cells was responsible for increased expression of inflammatory mediators and recruitment of innate immune cells during carcinogenesis; conversely, regulated TTP overexpression resulted in decreased skin infiltration of immune cells in this model (13). Wu et al. (32) reported that calcineurin inhibitors downregulate TTP expression in keratinocytes. In line with our data, this led to increased expression of inflammatory mediators in HRas^V12^-transformed keratinocytes. Malignant cell-derived TNF enhances the growth and spread of tumors in many different experimental models (33). However, the increased tumor development seen in *Zfp36*^ΔEP^** mice did not require keratinocyte-derived TNF production, indicating that TTP controls other pathways that play dominant roles in this model of tumorigenesis. As previously demonstrated in glioma cell lines (4), we could observe that neoplastic transformation of epidermal cells was accompanied by dysregulation of *Vegfa* expression, as well as that of *Pgf,* in the absence of TTP. This was associated with increased intratumoral angiogenesis. We also identified *Areg* mRNA as a direct target of TTP, and showed that inhibition of the EGFR pathway strongly decreased tumor burden in *Zfp36*^ΔEP^** mice. Of note, we also observed increased expression of *Ereg* (another EGFR ligand) and of *Adam12,* which acts as a “sheddase” for proHB-EGF (34). The role of the EGFR pathway in skin tumorigenesis is complex. Transgenic AREG overexpression in keratinocytes leads to inflammatory epidermal hyperplasia without spontaneous development of skin tumors, suggesting that aberrant EGFR signaling is not sufficient to drive tumorigenesis (35). Rather, EGFR signaling functions as a survival factor during oncogenic transformation (36). It is also critical for Ras-dependent VEGF induction and angiogenesis (37) and contributes to the release of IL-1α, leading to the activation of NF-κB, the production of CXCR2 ligands, and the suppression of keratinocyte differentiation (38). These results suggest that TTP activity in transformed cells could play a central role in reducing the oncogenic Ras/EGFR feed-forward loop that drives multiple aspects of tumor progression.

Ablation of TTP in hepatocytes was recently shown to decrease tumorigenesis upon diethylnitrosamine treatment, indicating that the effect of TTP on tumor initiation is context-dependent (39). It is possible that TTP plays a more important role in controlling tumor development at epithelial surfaces, as it is highly induced by inflammatory cues. We observed that expression of TTP was strongly heterogeneous in human malignant epidermal cells. Importantly, it was largely downregulated in a subset of TSKs that resides at the leading edges of the tumor in a fibrovascular niche (31). We can speculate that loss of TTP could contribute to the remodeling of the surrounding stroma through the regulation of key mediators known to promote angiogenesis (such as VEGF, PGF, or MMPs) or activate cancer-associated fibroblasts (such as activinβA; ref. 40). It would therefore be important to define the mechanisms leading to decreased *ZFP36*, *ZFP36L1*, and *ZFP36L2* expression during skin tumor progression.

In conclusion, we have demonstrated that ARE-mediated mRNA decay, specifically that aspect regulated by TTP, controlled key early steps of skin tumorigenesis in vivo. This pathway could therefore represent a valuable therapeutic target. More research is needed to understand the effects of TTP in the various aspects of tumor development identified in this study, as well as others still to be discovered.

## Methods

### Mice.

LoxP-flanked *Zfp36* mice (*Zfp36^fl/fl^)* mice on a C57BL/6 background were previously described (41). LysM-Cre (B6.129P2-*Lyz2*^tm1(cre)Ifo^), CD11c-Cre (B6.Cg-Tg(Itgax-Cre)1-1Reiz), and K14-Cre (Tg(KRT14-cre)1Amc) mice on C57BL/6 backgrounds were purchased from The Jackson Laboratory. Cell-specific *Zfp36*-deficient mice were generated by crossing the *Zfp36^fl/fl^* mice with mice expressing Cre recombinase under the control of the murine M lysozyme promoter (*Zfp36*^ΔM^**), the murine integrin alpha X promoter (*Zfp36*^ΔDC^**), or the human keratin 14 promoter (*Zfp36*^ΔEP^**). The double conditional *Zfp36*^ΔEP^*Tnf*^ΔEP^**mice were obtained by mating *Zfp36*^ΔEP^**mice with *Tnf^fl/fl^* mice, as previously described (11). *Zfp36*^ΔARE^ and *Zfp36-V5* knockin mice were previously described (13, 15). All experiments were performed using littermates as controls.

### DMBA/TPA 2-stage carcinogenesis.

For the experiments conducted with the *Zfp36*^ΔARE^ mice and corresponding controls, 7-week-old animals were anesthetized by inhalation of isoflurane, and the dorsal skin was shaved with surgical clippers and subsequently checked for the lack of hair growth. Initiation was accomplished by a single topical application of 400 nmol of DMBA in 200 μl acetone. Promoter treatments with 10 nmol of TPA in 200 μl acetone, twice a week, were begun 1 week after initiation and continued for 20 weeks. Skin tumors induced by the initiation-promotion protocol were counted.

For all the other experiments conducted, 8-week-old mice were anesthetized by i.p. injection of ketamine and xylazine and shaved on the back with an electrical shaver 2 days before DMBA treatment. Mice were treated on days 0 and 3 or 7 with 80 or 200 nmol of DMBA in 200 μl acetone. *Zfp36*^ΔEP^*, Zfp36*^ΔM^*,* and *Zfp36*^ΔDC^** mice were treated on days 0 and 3 with 200 nmol of DMBA in 200 μl acetone, and *Zfp36*^ΔEP^*Tnf*^ΔEP^** mice were treated on days 0 and 7 with 80 nmol of DMBA in 200 μl acetone. On day 14, mice were treated twice per week with 6.5 nmol of TPA in 200 μl acetone for 12 to 40 weeks. Tumor incidence and burden were assessed once per week. The experiment was stopped when the tumors reached greater than 10 mM. Mice were euthanized when indicated, and skin samples were prepared for immunofluorescence, gene expression, and flow cytometry analysis. When indicated, 500 μg/mL of AG1478 inhibitor in 200 μl acetone was applied 45 minutes before the TPA in 200 μl acetone, and 10 mg/kg of anti-TNF–blocking antibodies (XT3.11 clone, BioXcell) in PBS was injected i.p. 3 times a week from week 1 to week 10.

### TPA treatment.

The back skin of *Zfp36-V5* knockin mice and *Zfp36^fl/fl^* was shaved 2 days before the start of the experiment. Then, these mice were treated with 6.5 nmol of TPA in 200 μl acetone on the back skin once a day for 3 days. Skin biopsies were collected 24 hours later to perform flow cytometry analysis.

### Flow cytometry analysis.

Skin samples were incubated for 16 hours with dispase II (1 mg/mL, Sigma) and then with collagenase IV (1 mg/mL, Thermo Fisher) and DNAse I (100 μg/mL, Sigma) for 45 minutes at 37°C. Cells were stained for CD45-FITC (clone 30-F11) and γ/δ TCR-PerCP-eFluor710 (clone eBio-GL3) from eBioscience; Ly6G-PerCP-Cy5.5 (clone 1A8), IL-17A-APC (clone TC11-18H10), CD3 (clone 17A2), CD19 (clone 1D3), Gr1-APC-Cy7 (clone RB6-8C5), CD11b (clone M1/70), Ly6C (clone AL-21), and CD3-BV421 (clone 17A2) from BD Biosciences; and V5 Tag-AF647 (clone N/A, catalog 46-1260) from Invitrogen. Cells were incubated for 2 hours with PMA (25 ng/mL), ionomycin (500 ng/mL) and Brefeldin A (10 μg/mL) and processed for intracellular staining using the Intracellular Fixation and Permeabilization Kit (eBiosciences). Data were collected on a BD LSRII Fortessa and analyzed with FlowJoX software.

### FACS isolation of cells.

Tumors from mouse back skin were incubated for 2 hours with collagenase I (Roche, 3.5 mg/mL) on a rocking plate at 37°C. Enzyme activity was blocked by adding EDTA (5 mM). Cells were stained for CD45-PE (clone 30-F11) from eBioscience, CD140a-APC (clone APA5) and CD31-APC (clone MEC13.1) from BD Biosciences, and CD326-APC-Cy7 (clone G8.8) from Imtec. Living cells from tumors, DMBA/TPA-treated skin samples, and mock-treated skin samples were selected by the LIVE/DEAD Fixable Aqua Dead Cell Stain Kit exclusion (Invitrogen). Then, EpCAM (CD326)–positive cells were selected after CD140a, CD31, and CD45 exclusion. Sorting was performed on BD FACSAria II and analyzed with FACSDiva software.

### Cell culture.

Mouse primary keratinocytes were isolated from newborn mice (24–72 hours after birth) as described by Li et al. (42). Cells were maintained in complete Keratinocyte Growth Medium II (Promocell GmbH) at 36°C and 7% CO_2_. HEK293T cells (ATCC) were maintained in DMEM medium containing 10% FBS, 50 U/mL Penicillin, 50 μg/mL Streptomycin, and 1 mM sodium pyruvate (Gibco). Transfections were performed using the calcium phosphate method of transfection. Plasmid DNA was added to a CaCl_2_ solution, then was added dropwise into a Hepes-buffered phosphate solution. This DNA solution was then incubated at room temperature for 30 minutes before being spotted on the cell culture. Medium of the cells was changed prior to adding the DNA and after incubation of the cells overnight with the DNA mixture.

### Dual-reporter plasmids.

Plasmids containing the WT Globin 3′UTR (AU0 = no ARE) or Globin 3′UTR with AU-rich insertion (AUUU)_8_ (AU8 = canonical ARE sequence) were used as controls. The plasmids also contained the sequence coding for the firefly luciferase as a transfection efficiency control. Both luciferases were under the control of a bidirectional CMV promoter.

The WT 3′UTR of *Areg* gene was amplified by RT-PCR using the primers 5′‑agctagagcggccgcggatccCTGAGGACAATGCAGGGTAAA‑3′ and 5′gctcgaagcggccgcTGTTTAAAAAAAGTTTAATGAGCTATA‑3′ (lowercase letters indicate the leader sequences used for cloning), then cloned into the NotI site of the AU_0_ plasmid from Barreau et al.(30), using a ligation-independent cloning method. For *Areg*ΔARE, a DNA fragment containing the 3′UTR of *Areg* without the putative ARE motifs was synthesized (Integrated DNA Technologies), then amplified by PCR before cloning in the AU_0_ plasmid.

### Electrophoretic mobility shift assay.

Analysis of possible *Areg* mRNA-TTP interactions by supershift assay was performed as described in (43) with the following modifications. Cell extracts from HEK293T cells transfected with TTP-Flag of BOIP-Flag were incubated with ^32^P-labeled RNA probe, followed by the addition of anti-V5 or anti-Flag antibody. Areg probe corresponding to *Areg* 3’UTR AU-rich region (Figure 7B, underlined sequence) and control probe corresponding to pBS polylinker region were produced by in vitro transcription using T7 RNA polymerase and ^p32^UTP. Samples were loaded on 5% polyacrylamide nondenaturing gels containing 6% glycerol and 0.5× TBE at 7.5 mA for 16 hours at 4°C. Competition experiments were performed by adding 2-, 4-, 8-, 16-, or 32-fold molar excess of unlabeled Areg probe or control pBS probe.

### Gene expression (2-step qPCR).

Total RNA was extracted with the RNeasy Mini Kit (Qiagen) and reverse-transcribed with the High-Capacity cDNA Archive Kit (Applied Biosystems). cDNA was amplified using SYBR green or TaqMan probes. Primer sequences are described in Supplemental Table 1.

### Immunofluorescence staining.

Tumors from mouse tissues were embedded and frozen in OCT (Tissue-Tek). Sections (6 μm) were stained as explained in ref. 44. Anti-Endoglin (CD105, polyclonal, R&D), anti-Keratin 14 (clone SIG-3476, Thermo Fisher), and anti-VE-Cadherin (CD144, clone 11D4.1, BD Bioscience) were used as primary antibodies. The following secondary antibodies were used: anti-goat, anti-rat, anti-rabbit conjugated to AlexaFluor488 (Molecular Probes), to rhodamine Red-X (Jackson ImmunoResearch), or to Cy5 (Jackson ImmunoResearch), respectively. Nuclei were stained in Hoechst solution. Slides were observed at 200-fold magnification. Images were acquired using Zeiss AxioImager M1 and analyzed with Zen 2.3 lite (blue edition) software.

### RNA-Seq.

For the RNA-Seq analysis of skin biopsies from the *Zfp36*^ΔARE^ mice and their corresponding controls, full-thickness skin biopsies were taken at the end of the experiment. These were all from treated but nontumoral skin. Total RNA was isolated by pestle homogenization using a TRIzol and chloroform method according to the manufacturer’s instructions (Invitrogen). Raw pair-end fastq files were provided by the sequencing contractor (https://www.Q2Labsolutions.com). Low-quality sequence reads with a mean score less than 20 were removed using a custom perl script. The processed reads were mapped to the mm10 genome using the Spliced Transcripts Alignment to a Reference software (v2.5.2b) (45). The number of fragments per gene were counted using the featureCounts command available from Subread (v1.5.0-p1) (46). Differential gene expression analysis was performed using the R package DESeq2 (version 1.12.4) (47).

Total RNA from sorted epidermal cells (isolated from mock-treated skin, DMBA/TPA-treated adjacent skin, and papillomas) was extracted using an RNeasy Plus Micro Kit (Qiagen). Quality control, library preparation, and RNA-Seq were performed by BRIGHTcore ULB VUB (http://www.brightcore.be). RNA-Seq was performed on triplicates of each group, using the standard Illumina HiSeq sequencing protocol (20 × 10^6^ reads/sample). FastQC was used for read quality determination. Differential gene expression analysis was done using the EdgeR method, with FDR less than 0.05 and fold change as indicated.

ARE score analysis was performed according to the scoring option (http://arescore.dkfz.de/arescore.pl) (17). We first defined a list of genes differentially expressed in tumors from *Zfp36*^ΔEP^** mice compared with tumors from *Zfp36^fl/fl^* mice (minimal read count of 10 in RNA-Seq). We extracted all isoforms of described transcripts from the RefSeq database.

### scRNA-Seq analysis.

We retrieved single-cell transcriptomic profiles of 7 normal skin and 7 cSCC from a recently published data set (31) (accession GSE144236). We restricted our analysis to epithelial cells, including normal keratinocytes (basal, cycling, and differentiating) and tumor keratinocytes (basal, cycling, and differentiating and TSKs) subpopulations, to obtain a total of 18,359 cells. A Seurat object was created using Seurat r package v3.2.2 and cells-metadata, established by the authors (31), was added by applying *AddMetaData* function. Counts were normalized with SCTransform method by which we regressed mitochondrial-mapped genes and UMI counts. We ran the dimensionality reduction functions (*RunPCA* and *RunUMAP*) with the first 15 PCs as input. A shared nearest neighbor graph and Seurat clusters were identified. Cluster annotation was done according to the added cells-metadata. To visualize the reduced dimension coordinates of the annotated cells, we used the *DimPlot* function, and gene expression was assessed using *FeaturePlot* and *VlnPlot* functions. To determine the enrichment of DEGs in WT or *Zfp36*^ΔEP^** tumor cells obtained from our bulk RNA-Seq data, 2 gene lists were created, and enrichment score was measured by applying the *AddModuleScore* Seurat function.

### Data availability.

Raw RNA-Seq data can be accessed on GEO (accession GSE148199 and GSE151587).

### Statistics.

Results are expressed as mean ± SEM. The statistical significance was assessed as indicated using GraphPad Prism 8.0. The following tests were used: Mantel-Cox log rank test, 1-way or 2-way ANOVA test with Bonferroni’s correction or post hoc Tukey’s HSD test, and 2-tailed Mann-Whitney test. A *P* value less than 0.05 was considered significant.

### Study approval.

Animal studies performed in Belgium were approved by the institutional animal care and local committee for animal welfare of the BIOPOLE ULB CHARLEROI. Animal studies performed at the National Cancer Institute (NCI) were approved by the IACUC of the NCI (Frederick, Maryland, USA) and were conducted in accordance with the IACUC guidelines and the *Guide for the Care and Use of Laboratory Animals* (National Academies Press, 2011). Animals were maintained in a specific pathogen–free facility with ad libitum access to water and feed. All animals were used in scientific experiments for the first time.

## Author contributions

A. Assabban, IDV, and LVM conducted most of the experiments. RS, BS, LZ, A. Azouz, CL, MM, CG, and SW contributed to some experiments; MN and ST provided technical help for the experiments. A. Azouz, BDT, and LZ performed transcriptomic analysis. A. Assabban analyzed the data and prepared the figures. CB, CG, VK, GT, and GL provided input for research design and interpretation. WH provided critical reagents. SG and PJB supervised the work and wrote the manuscript. All authors were involved in critically revising the manuscript for important intellectual content. All authors had full access to the data and approved the manuscript before it was submitted by the corresponding author.

## Supplementary Material

Supplemental data

## Version 1 01/26/2021

In-Press Preview

## Version 2 03/08/2021

Electronic publication
