# Supplementary material for: Tristetraprolin expression by keratinocytes protects against skin carcinogenesis
Source: JCI Insight. 2021 Mar 8;6(5):e140669. doi: 10.1172/jci.insight.140669 (PMC8021119; doi:10.1172/jci.insight.140669)
Supplement: Supplemental data [file jciinsight-6-140669-s232.pdf]

Tristetraprolin expression by keratinocytes protects against skin carcinogenesis

Assabban *et al*, Supplementary figures

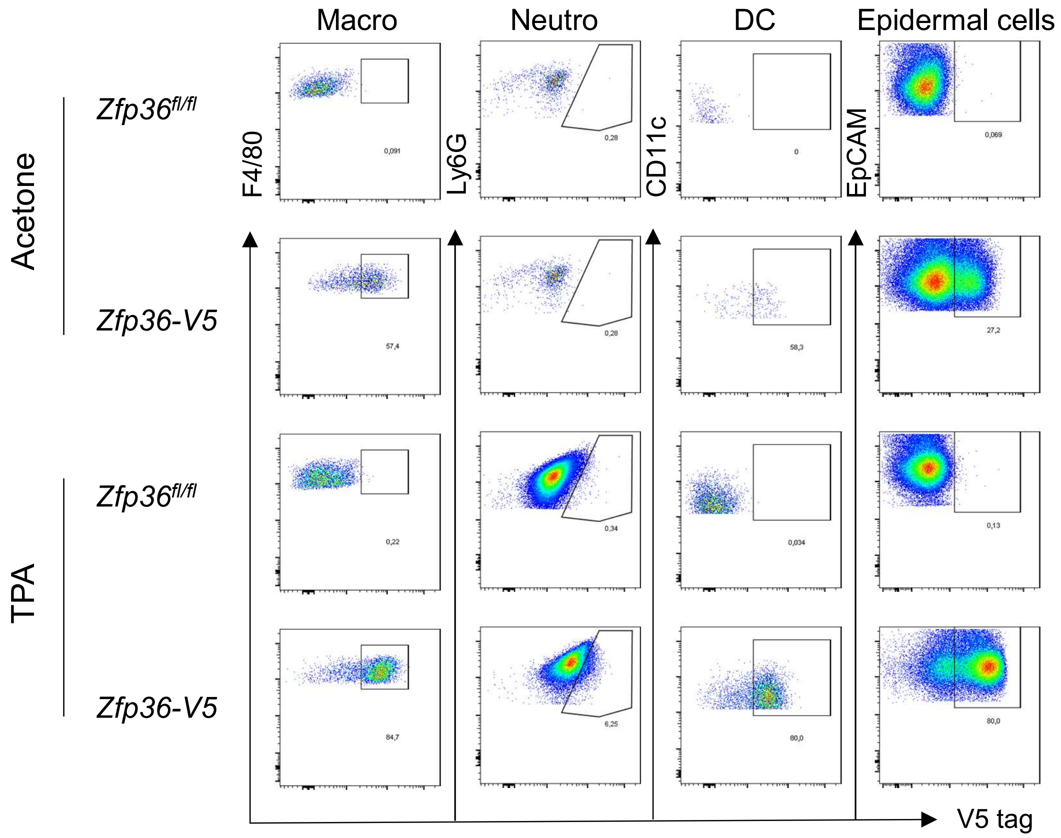

Figure S1 (related to Figure 2A): Gating strategies for skin cell populations after acute inflammatory stimulation. *Zfp36-V5* and *Zfp36$^{fl/fl}$* mice were treated for 3 days with TPA or acetone. Representative skin immune cells were gated based on the following gating strategy: MHCII^{high} macrophages (CD45^{+}CD19^{-}CD3ε^{-}CD11b^{+}Ly6G^{-}F4/80^{+}MHCII^{high}), neutrophils (CD45^{+}CD19^{-}CD3ε^{-}CD11b^{+}Ly6G^{+}) and DCs (CD45^{+}CD19^{-}CD3ε^{-}CD11b^{+}Ly6G^{-}F4/80^{-}CD11c^{+}MHCII^{+}). Keratinocytes were gated based on CD45^{-}CD140a^{-}CD31^{-}EpCAM^{+} staining.

Neutrophils

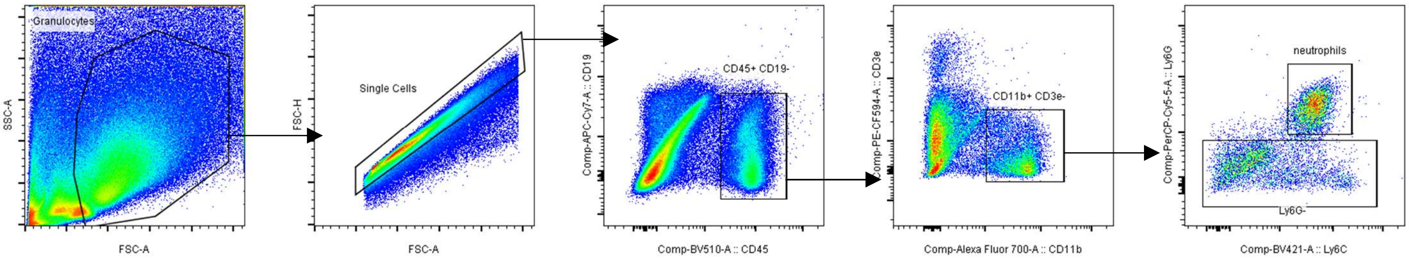

γδT cells

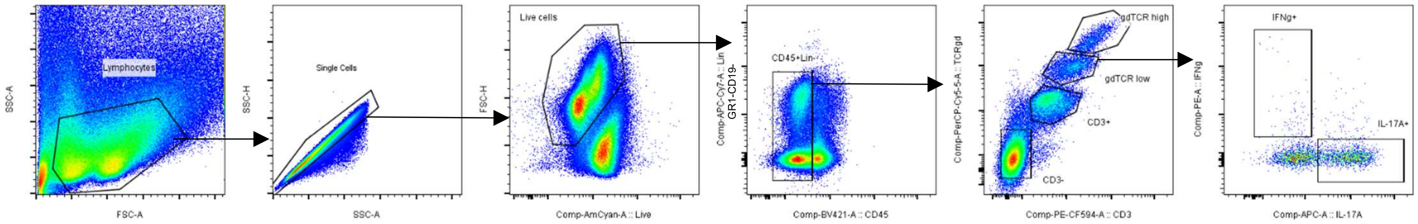

Figure S2 (related to Figure 2D): Gating strategies for skin immune cell populations after DMBA/TPA treatment. *Zfp36*$^{\Delta EP}$ mice and their littermate controls (*Zfp36*$^{fl/fl}$) were topically treated on back skin with DMBA/TPA for 12 to 20 weeks. After exclusion of doublets, neutrophils were characterized based on CD19^{-}CD3ε^{-}CD45^{+}CD11b^{+}Ly6C^{+}Ly6G^{hi}. After exclusion of dead cells, IL17A-producing dermal γδ T cells were selected by GR1^{-}CD19^{-}CD45^{+}CD3ε^{+}γδTCR^{+}IL17A^{+} staining.

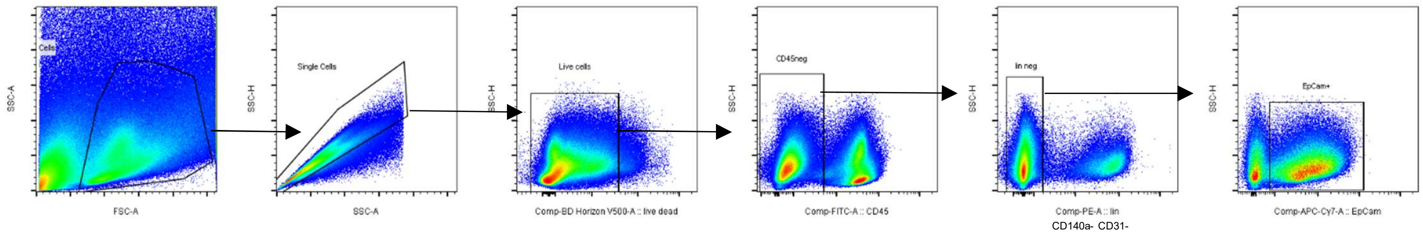

Figure S3 (related to Figure 4): Gating strategy to isolate EpCAM-positive cells from skin and tumors after DMBA/TPA treatment. After up to 40 weeks of carcinogenic treatment on *Zfp36*$^{\Delta EP}$ and control mice, epithelial cells from papillomas (~3 mm size), adjacent non-tumoral skin and mock skin, were sorted by exclusion of dead cells and CD45^{-}CD140a^{-}CD31^{-}EpCAM^{+} selection.

Supplementary table

Table S1: List of RT-PCR primers

Target	Sequence FW 5' to 3'	Sequence RV 5' to 3'	Taqman probe 5' to 3'
Actb	TCC-TGA-GCG-CAA-GTA-CTC-TGT	CTG-ATC-CAC-ATC-TGC-TGG-AAG	ATC-GGT-GGC-TCC-ATC-CTG-GC
Areg	GGA-CAA-TGC-AGG-GTA-AAA-GTT-	TGA-AAG-AAG-GAC-CAA-TGT-CAT-	/
Cxcl1	CCG-AAG-TCA-TAG-CCA-CAC-TC	TTT-CTG-AAC-CAA-GGG-AGC-TT	AAG-GCA-AGC-CTC-GCG-ACC-AT
Cxcl2	ACA-TCC-AGA-GCT-TGA-GTG-TGA	GCC-CTT-GAG-AGT-GGC-TAT-G	CCC-ACT-GCG-CCC-AGA-CAG-AA
Il17f	ATT-CCA-GAA-CCG-CTC-CAG-TT	CTG-GGC-CTC-AGC-GAT-CTC-T	TGG-GAT-TAC-AAC-ATC-ACT-CGA-GAC-CC
Inhba	GGA-GAA-CGG-GTA-TGT-GGA-GA	TGG-TCC-TGG-TTC-TGT-TAG-CC	/
Lcn2	GAC-TTC-CGG-AGC-GAT-CAG-T	ACA-TCG-TAA-AGC-TGC-CTT-CTG	TGG-ACC-GCA-TTG-CCT-GCCAGG-CCC-A
Lif	AGA-ATC-AAC-TGG-CAC-AGC	TGG-AAA-GAT-GGG-AAG-TCT-G	/
Pgf	CCG-GCC-CTG-GCT-GCA-TTG-AA	CAG-GCA-AAG-CCC-ACA-GGC-GA	/
S100a8	CCT-TTG-TCA-GCT-CCG-TCT-TC	CAA-GGC-CTT-CTC-CAG-TTC-AG	AAG-GAA-ATC-TTT-CGT-GACAAT-GCC-G
S100a9	AGC-CTT-GAG-CAA-GAA-GAT-GG	TTG-ATG-GAA-GGT-GTC-GAT-GA	TGG-AGC-GCA-GCA-TAA-CCA-CCA
Sema4d	CCG-ACC-TGG-AGC-GTG-TAT-C	AGA-CAA-ACT-TCC-TCC-CCT-TC	/
Sema6d	CAG-AAG-CAT-GGG-AGA-TGG-AT	GCC-ACC-CAT-GTC-GTT-TTT-AC	/
Tnf	CAG-ACC-CTC-ACA-CTC-AGA-TCA	CAC-TTG-GTG-GTT-TGC-TAC-GA	TCG-AGT-GAC-AAG-CCT-GTA-GCC-CA
Zfp36	GAC-CAC-CGG-ACA-CTG-AAC-T	TCC-TGG-AAT-CTT-AAG-TGCTGT-GA	/
